# Aberrantly Expressed lncRNAs and mRNAs of Osteogenically Differentiated Mesenchymal Stem Cells in Ossification of the Posterior Longitudinal Ligament

**DOI:** 10.3389/fgene.2020.00896

**Published:** 2020-08-07

**Authors:** Zhaopeng Cai, Wenjie Liu, Keng Chen, Peng Wang, Zhongyu Xie, Jinteng Li, Ming Li, Shuizhong Cen, Guiwen Ye, Zhaofeng Li, Zepeng Su, Mengjun Ma, Yanfeng Wu, Huiyong Shen

**Affiliations:** ^1^Department of Orthopedics, The Eighth Affiliated Hospital, Sun Yat-sen University, Shenzhen, China; ^2^Department of Orthopedics, Sun Yat-sen Memorial Hospital, Sun Yat-sen University, Guangzhou, China; ^3^Center for Biotherapy, Sun Yat-sen Memorial Hospital, Sun Yat-sen University, Guangzhou, China

**Keywords:** ossification of the posterior longitudinal ligament, long non-coding RNA, mesenchymal stem cells, osteogenic differentiation, mRNA

## Abstract

Ectopic bone formation is the chief characteristic of ossification of the posterior longitudinal ligament (OPLL). Emerging evidence has revealed that long non-coding RNAs (lncRNAs) can regulate the osteogenic differentiation of mesenchymal stem cells (MSCs), which are the main cells responsible for bone formation. However, the role of lncRNAs in the pathogenesis of OPLL remains unclear. In this study, 725 aberrantly expressed lncRNAs and 664 mRNAs in osteogenically differentiated MSCs from OPLL patients (OPLL MSCs) were identified by microarrays and confirmed by qRT-PCR assays. Gene Ontology (GO) and Kyoto Encyclopedia of Genes and Genomes (KEGG) pathway analyses showed that the most enriched pathways included the p53, JAK-STAT, and PI3K-Akt signaling pathways. The co-expression network showed the interactions between the aberrantly expressed lncRNAs and mRNAs in OPLL MSCs, and the potential targets and transcription factors of the lncRNAs were predicted. Our research demonstrated the aberrantly expressed lncRNA and mRNA and the potential regulatory networks involved in the ectopic bone formation of OPLL. These findings imply that lncRNAs may play a vital role in OPLL, which provides a new perspective on the pathogenesis of OPLL.

## Introduction

Ossification of the posterior longitudinal ligament (OPLL) is a common disease characterized by the ectopic bone formation of spinal ligaments (Xu et al., 2016; Yan et al., 2017). In the past several decades, studies have revealed many factors contributing to the development of OPLL, including diabetes and genetic, environmental, hormonal and lifestyle factors (Nakajima et al., 2014; Nakajima et al., 2016; Chen et al., 2018). However, the pathogenesis of OPLL remains unclear. The ossified ligaments enlarge over time and eventually compress the spinal cord, causing serious neurological problems (Tanaka et al., 2011; Xu et al., 2018). There are no effective therapies for preventing the formation and progression of ossified ligaments other than surgery (Shi et al., 2019). Therefore, it is of great significance to elucidate the pathogenesis of OPLL and look for new therapeutic targets for OPLL.

Mesenchymal stem cells (MSCs) can be isolated from a variety of other tissues, including umbilical cord blood, dental pulp, adipose tissue, tendon, skin and muscle, but they are mainly derived from bone marrow (Ding et al., 2011; Bernardo and Fibbe, 2013). MSCs can differentiate into osteoblasts, which are responsible for bone formation (Crane and Cao, 2014). In recent years, MSCs have been demonstrated to be involved in the pathogenesis of many diseases, including fibrotic diseases, systemic lupus erythematosus, ankylosing spondylitis and osteoporosis (Tan et al., 2015; Xie et al., 2016b; El Agha et al., 2017; Wu et al., 2018; Geng et al., 2019; Liu w. et al., 2019). Recent studies suggested an increase in the osteogenic potential of MSCs from OPLL patients, which may be a pathogenic factor of OPLL (Harada et al., 2014; Liu X. et al., 2017). However, the concrete mechanism underlying the role of MSCs in the abnormal osteogenic differentiation of OPLL is less known.

Long non-coding RNAs (lncRNAs) are defined as transcripts that are more than 200 nucleotides in length that do not encode proteins (Chen et al., 2017). The non-coding genome was previously considered junk DNA (Kopp and Mendell, 2018). However, with the development and application of high-throughput technologies, it is surprising that more than 98% of the human genome does not encode proteins (Kopp and Mendell, 2018). Many studies have demonstrated that lncRNAs play an important role in cell proliferation, differentiation and apoptosis (Li M. et al., 2018; Tang S et al., 2018). Recent studies have shown that lncRNAs are involved in the occurrence and progression of many diseases, such as non-small cell lung cancer, hepatocellular carcinoma, and ankylosing spondylitis (Xie et al., 2016a, 2018; Huang et al., 2019). However, little is known about the functions of lncRNAs in the pathogenesis of OPLL.

In this study, aberrantly expressed lncRNAs and mRNAs in osteogenically differentiated MSCs from OPLL patients (OPLL MSCs) were detected by microarrays and confirmed by qRT-PCR assays. Gene Ontology (GO) and Kyoto Encyclopedia of Genes and Genomes (KEGG) pathway analyses were performed to identify the cellular events and biological pathways involved in the abnormal osteogenic differentiation of OPLL MSCs. The co-expression network showed the interactions between the aberrantly expressed lncRNAs and mRNAs in OPLL MSCs, and the potential targets and transcription factors of the lncRNAs were predicted. Our findings provide a new perspective on the mechanisms of the ectopic bone formation of spinal ligaments in OPLL.

## Materials and Methods

### Cell Isolation and Culture

In this study, 30 healthy donors (HDs) and 30 OPLL patients were recruited. The OPLL was diagnosed based on clinical examinations including cervical radiographs, computed tomography (CT), and magnetic resonance imaging (MRI). Details of the study subjects were presented in Supplementary Table S1. The study was approved by the Ethics Committee of Sun Yat-sen Memorial Hospital (Sun Yat-sen University, Guangzhou, China), and informed consent was obtained from all participants. MSCs were isolated from bone marrow and cultured in Dulbecco’s modified Eagle’s medium (DMEM; Gibco) containing 10% fetal bovine serum (FBS; Sijiqing Biological Engineering Material Company). All cells were cultured in 5% CO_2_ at 37°C, and the culture medium was changed every 3 days.

### Flow Cytometry

MSCs were digested with 0.25% trypsin containing 0.53 mM EDTA and washed thoroughly with phosphate-buffered saline (PBS). After incubation with specific antibodies for 30 min, the MSCs were washed again in preparation for flow cytometry with a BD Biosciences Influx cell sorter (BD Biosciences). Antibodies against human phycoerythrin (PE)-conjugated CD105, PE-conjugated CD73, PE-conjugated CD 90, fluorescein isothiocyanate (FITC)-conjugated CD45, FITC-conjugated CD34, FITC-conjugated CD14, FITC-conjugated 79-a, and FITC-conjugated HLA-DR were used in the study (all from BD PharMingen).

### Osteogenic Differentiation

To induce osteogenic differentiation, MSCs (1.5 × 10^4^ cells/cm^2^) were seeded in 12-well plates and cultured in osteogenic differentiation medium containing DMEM with 10% FBS, 100 IU/ml penicillin, 100 IU/ml streptomycin, 0.1 μM dexamethasone, 10 mM β-glycerol phosphate and 50 μM ascorbic acid (Sigma-Aldrich, St. Louis, MO, United States). The medium was changed every 3 days.

### Alkaline Phosphatase (ALP) Activity and Staining

To detect ALP activity, the cells were cultured in osteogenic differentiation medium for 10 days. Protein was extracted from the cells using RIPA buffer (Sigma), and the protein concentration was determined with the Pierce BCA Protein Assay Kit (Thermo Fisher Scientific). Then, ALP activity was detected using an ALP activity kit (Nanjing Jiancheng Biotech, Nanjing, China) according the manufacturer’s protocol. For ALP staining, the cells were fixed and stained using the BCIP/NBT Alkaline Phosphatase Color Development Kit (Beyotime) according the manufacturer’s protocols.

### Alizarin Red S (ARS) Staining and Quantification

To perform ARS staining, the cells were cultured in osteogenic differentiation medium for 14 days. The cells were fixed with 4% paraformaldehyde and stained with 1% ARS for 15 min. After washing thoroughly with PBS, the cells were observed under a microscope. For ARS quantification, the above cells were destained using 10% cetylpyridinium chloride (CPC) monohydrate (Sigma-Aldrich). Then, 200 μl of supernatant was taken from the above mixture and transferred to a 96-well plate to measure the absorbance at 562 nm.

### Real-Time Quantitative Reverse Transcription Polymerase Chain Reaction (qRT-PCR)

After adding TRIzol (Invitrogen, Massachusetts, United States), total RNA was extracted from the cells. Then, cDNA was transcribed from 1 μg of RNA with the PrimeScript RT Reagent Kit (TaKaRa, Dalian, China) according to the manufacturer’s instructions. qRT-PCR was performed with the LightCycler^®^480 PCR system (Roche, Basel, Switzerland) using SYBR Premix Ex Taq (TaKaRa) according to the manufacturer’s protocol. GAPDH was used as the internal reference, and the relative expression levels of all genes were calculated using the 2^−ΔΔCt^ method. The primer sequences are shown in Supplementary Table S2.

### RNA Isolation and Microarray Analysis

The cells used for microarray analysis were cultured in osteogenic differentiation medium for 10 days. After adding TRIzol (Invitrogen), total RNA was extracted from three HDMSC samples (control group; sample H1–H3) and three OPLLMSC samples (experimental group; sample O1–O3). RNA integrity was detected by 1% formaldehyde denaturing gel electrophoresis. Using the CbcScript reverse transcriptase with cDNA synthesis system, double-stranded cDNAs (dsDNAs) were synthesized from 1 μg RNA according to the manufacturer’s instruction (CapitalBio). Then the dsDNA products were purified with a PCR NucleoSpin Extract II Kit (MN) after completion of dsDNA synthesis using DNA polymerase and RNase H. The dsDNA were amplified by PCR and purified using the RNA Clean-up Kit (MN). Finally, microarrays (CapitalBio Co.) were performed according to the manufacturer’s protocol.

The microarray data were analyzed using GeneSpring software V13.0 (Agilent). To identify the differentially expressed genes, genes with fold changes ≥ 2 and *p* < 0.05 were considered significant. The data were log2 transformed and median centered (by genes) using the Adjust Data function of CLUSTER 3.0 software and then further analyzed with hierarchical clustering with average linkage.

### GO and KEGG Pathway Analyses

GO analysis was performed to provide a label classification for gene function and gene product attributes in three domains (cellular component, molecular function and biological process) using KOBAS 3.0 software. KEGG pathway analysis was performed to identify the significant enrichment of different pathways using KOBAS 3.0 software.

### Construction of the Co-expression Network

Based on the correlations between the differentially expressed lncRNAs and mRNAs, a co-expression network was constructed. The Pearson correlation for each pair of genes was calculated, and the significant correlation pairs were selected to construct the network using Cytoscape software. Pearson correlation coefficient ≥ 0.99 was considered significant.

### Target Prediction of lncRNAs

The target prediction included *cis-*acting lncRNA prediction and trans-acting lncRNA prediction. The cis-acting lncRNA prediction was performed by assessing the close correlation of the lncRNA (Pearson correlation coefficient ≥ 0.99) to coding genes. The lncRNA must reside at genomic loci where a coding gene and the lncRNA gene were within 10 kb of each other along the genome. The trans-prediction was carried out using BLAT tools (Standalone BLAT v. 35 × 1) to compare the full sequence of the lncRNA with the 3′ untranslated region (UTR) of its co-expression mRNAs.

### Transcription Factor Prediction of lncRNAs

Transcription factor prediction was performed using the Match-1.0 Public transcription factor prediction tool. Based on the results of the co-expression network, the binding of the upstream 2000 bp region and the downstream 500 bp region of the starting site of lncRNAs with transcription factors was predicted.

### Statistical Analysis

Statistical analysis was performed with SPSS 22.0 software (Chicago, IL, United States). Student’s *t*-test was used to compare the differences between two groups. A value of *p* < 0.05 was considered significant.

## Results

### Phenotypes and Osteogenic Differentiation Capacities of HD MSCs and OPLL MSCs

**FIGURE 1:**
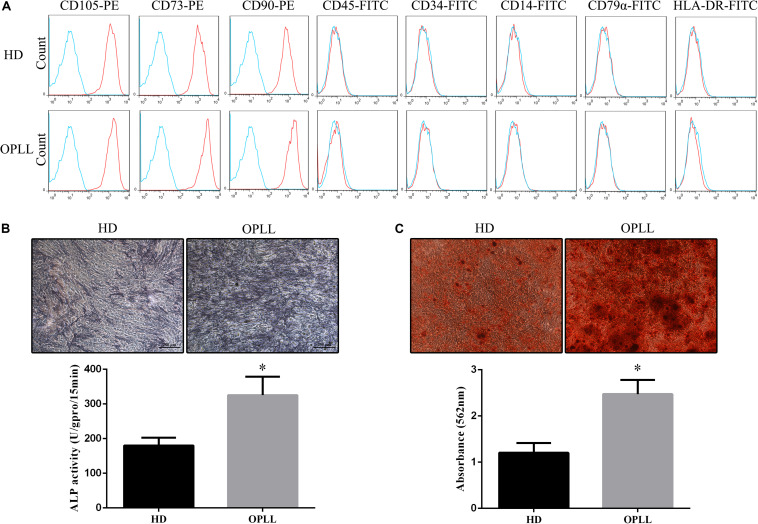
Phenotypes and osteogenic differentiation capacities of HD MSCs and OPLL MSCs. **(A)** Both HD MSCs and OPLL MSCs expressed typical MSC surface markers, with positivity for CD105, CD73, and CD90 and negativity for CD45, CD34, CD14, CD79a, and HLA-DR. **(B)** Alkaline phosphatase (ALP) assays were performed to assess the osteogenic differentiation capacity of MSCs. OPLL MSCs showed greater ALP staining than HD MSCs after 10 days of induction. The absorbance of ALP quantification of OPLL MSCs was also greater than that of HD MSCs. **(C)** Alizarin red S (ARS) staining assays were performed to assess the osteogenic differentiation capacity of MSCs. OPLL MSCs showed greater ARS staining than HD MSCs after 14 days of induction. The absorbance of ARS quantification of OPLL MSCs was also greater than that of HD MSCs. *n* = 15. ^∗^*p* < 0.05. HD MSC: mesenchymal stem cells from healthy donors; OPLL MSC: MSCs from patients with ossification of the posterior longitudinal ligament.

Both HD MSCs and OPLL MSCs expressed typical MSC surface markers, with positivity for CD105, CD73, and CD90 and negativity for CD45, CD34, CD14, CD79a, and HLA-DR (Figure 1A). ALP staining assays showed that the ALP activity of OPLL MSCs was stronger than that of HD MSCs after osteogenic differentiation (Figure 1B). In addition, OPLL MSCs showed greater ARS staining than HD MSCs after osteogenic differentiation (Figure 1C). These results indicate that OPLL MSCs have a greater capacity for osteogenic differentiation than HD MSCs.

### Expression Profiles of lncRNAs and mRNAs in Osteogenically Differentiated OPLL MSCs Compared to HD MSCs

**FIGURE 2:**
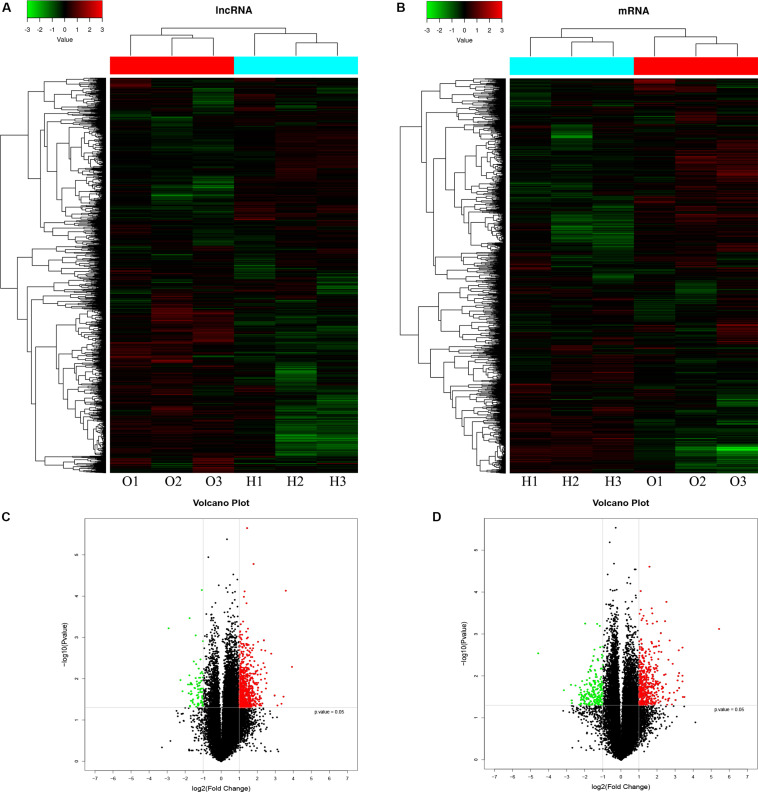
LncRNA and mRNA expression profiles in osteogenically differentiated OPLL MSCs compared to HD MSCs. Cluster heat map shows differentially expressed lncRNAs **(A)** and mRNAs **(C)** in OPLL MSCs. The names of the sample groups are on the x-axis, and the different probes are on the y-axis. The red strip indicates high relative expression, and the green strip indicates low relative expression. O1–O3: group with OPLL MSCs; H1–H3: group with HD MSCs. Volcano plots of lncRNAs **(B)** and mRNAs **(D)** in OPLL MSCs vs. HD MSCs. The red squares in the plots represent the upregulated transcripts with statistical significance, while the green squares represent the downregulated transcripts (fold change ≥ 2.0, *p* < 0.05).

A microarray was used to identify differentially expressed lncRNAs and mRNAs between osteogenically differentiated OPLL MSCs and HD MSCs, and a total of 28,156 lncRNAs and 28,339 mRNAs expressed in MSCs were detected. Hierarchical clustering analysis showed homogeneous lncRNA and mRNA expression profiles within the same group and distinct expression profiles between the two groups (Figures 2A,C). Compared to that in osteogenically differentiated HD MSCs, in OPLL MSCs, 725 lncRNAs were differentially expressed, including 651 upregulated lncRNAs and 74 downregulated lncRNAs (fold change > 2.0, *P* < 0.05) (Figure 2B). In addition, 664 mRNAs were differentially expressed in osteoclasts, including 453 upregulated mRNAs and 211 downregulated mRNAs (fold change > 2.0, *P* < 0.05) (Figure 2D). The top 10 differentially expressed lncRNAs and mRNAs are shown in Tables 1, 2, respectively.

**TABLE 1:** Top 10 differentially expressed lncRNAs from microarray data (HD vs. OPLL).

**Probe Name**	**Gene symbol**	**Chromosome**	**Start**	**End**	**Genome Relationship**	**Expression**	**Fold change**	***P*-value**
p11878	ENST00000470263.1	3	82035288	82512826	Intergenic	up	15.245	0.005
p23569	TCONS_00012620	6	158137089	158210465	Intergenic	up	11.953	0.000
p22941	TCONS_00009244	5	27472390	27494442	Intergenic	up	10.934	0.027
p20197	TCONS_00025725	17	59470837	59477096	Divergent	up	10.172	0.040
p13631	ENST00000512067.1	5	27472398	27486253	Intergenic	up	8.676	0.044
p13632	ENST00000510165.1	5	27472398	27496508	Intergenic	up	7.804	0.025
p26842	HIT000323653	5	83016570	83017020	Antisense	down	7.581	0.001
p1957	ENST00000422842.1	10	56245989	56415811	Intronic	up	6.833	0.002
p8129	ENST00000567905.1	19	2727740	2729325	Sense	up	5.723	0.007
p4982	ENST00000557226.1	14	100800126	100820398	Antisense	up	5.701	0.012

**TABLE 2:** Top 10 differentially expressed mRNAs from microarray data (HD vs. OPLL).

**Gene Symbol**	**Seq name**	**Expression**	**Fold change**	***P*-value**
CST2	NM_001322	up	42.950	0.001
HAPLN1	NM_001884	down	23.922	0.003
CLSTN2	NM_022131	up	10.756	0.032
NDUFA4L2	NM_020142	up	10.586	0.010
CST1	NM_001898	up	10.575	0.002
OLFM2	ENST00000264833	up	10.563	0.014
CYP26B1	NM_019885	up	9.359	0.019
ADH1C	NM_000669	up	9.317	0.009
ADH1B	ENST00000305046	up	9.004	0.010
MMP13	NM_002427	down	8.929	0.022

### Validation of the Expression Profiles by qRT-PCR

**FIGURE 3:**
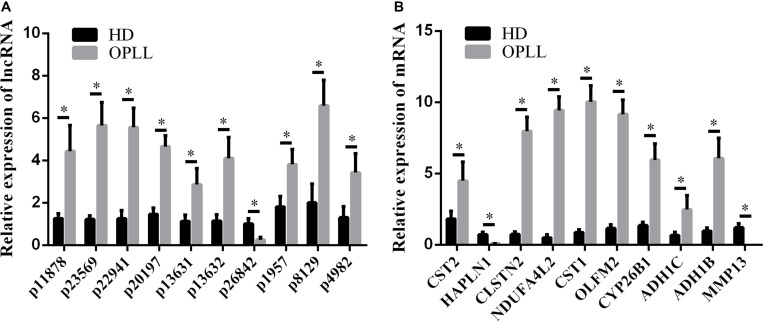
Validation of the expression profiles by qRT-PCR. Several genes were selected for the qRT-PCR assays. **(A)** Several lncRNAs were selected for the qRT-PCR assays. **(B)** Several mRNAs were selected for the qRT-PCR assays. The data are presented as the mean ± SD (*n* = 30). The results represent three independent experiments. ^∗^*p* < 0.05.

To confirm the microarray data, the top 10 differentially expressed lncRNAs and mRNAs were selected for the qRT-PCR assays. Their expression levels in HD MSCs and OPLL MSCs are shown in Figures 3A,B. For the lncRNAs, p11878, p23569, p22941, p20197, p13631, p13632, p1957, p8129 and p4982 were upregulated in OPLL MSCs compared to HD MSCs, while p26842 was downregulated. For the mRNAs, CST2, CLSTN2, NDUFA4L2, CST1, OLFM2, CYP26B1, ADH1C, and ADH1B were upregulated in OPLL MSCs compared to HD MSCs, while HAPLN1 and MMP13 were downregulated. These results of the qRT-PCR assays were almost consistent with the microarray data (Supplementary Table S3).

### GO and KEGG Pathway Analyses

**FIGURE 4:**
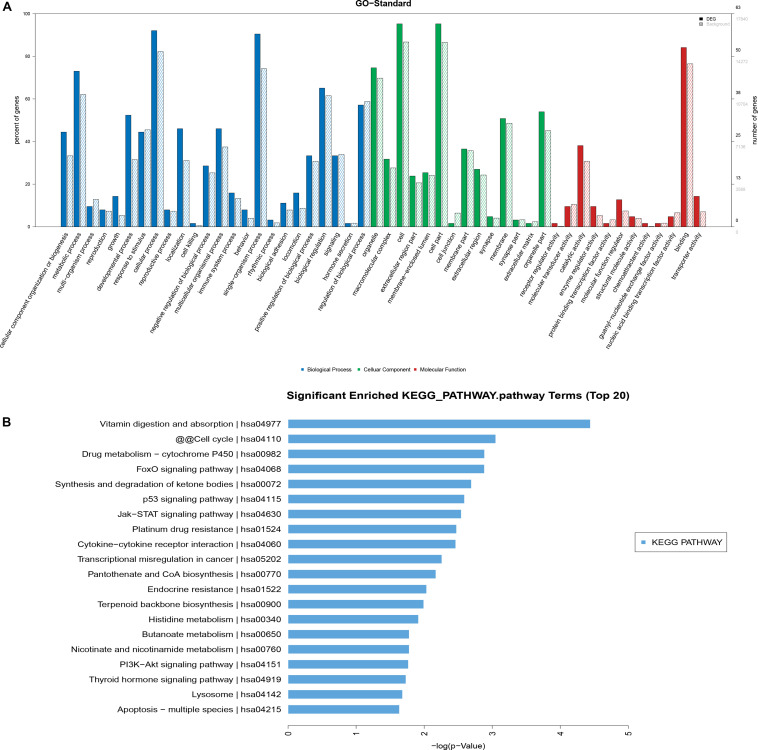
GO and KEGG pathway analyses. **(A)** The GO terms with significantly differential expression from GO analysis of the biological process, cellular component and molecular function categories are shown. **(B)** The top 20 KEGG pathways with significantly differential expression are shown. Higher -log P (LgP) values indicate higher significance, and lower -LgP values indicate lower significance.

The differentially expressed mRNAs were used in GO analysis. A total of 2,156 GO terms in the biological process category were differentially expressed between HD MSCs and OPLL MSCs (*P* < 0.05), including cellular component organization or biogenesis (GO: 0071840). There were 249 GO terms in the cellular component category that were differentially expressed between HD MSCs and OPLL MSCs (*P* < 0.05), including organelles (GO: 0043226). A total of 372 GO terms in the molecular function category were differentially expressed between HD MSCs and OPLL MSCs (*P* < 0.05), including receptor regulator activity (GO: 0030545). The significantly enriched GO terms are presented in Figure 4A.

KEGG pathway analysis showed that 119 pathways were statistically significantly enriched. The most enriched pathways included the p53, JAK-STAT and PI3K-Akt signaling pathways (Figure 4B), suggesting that these signaling pathways may play important roles in the osteogenic differentiation of OPLL MSCs. The top 20 significantly enriched pathways are presented in Figure 4B.

### LncRNA-mRNA Co-expression Network Analysis

**FIGURE 5:**
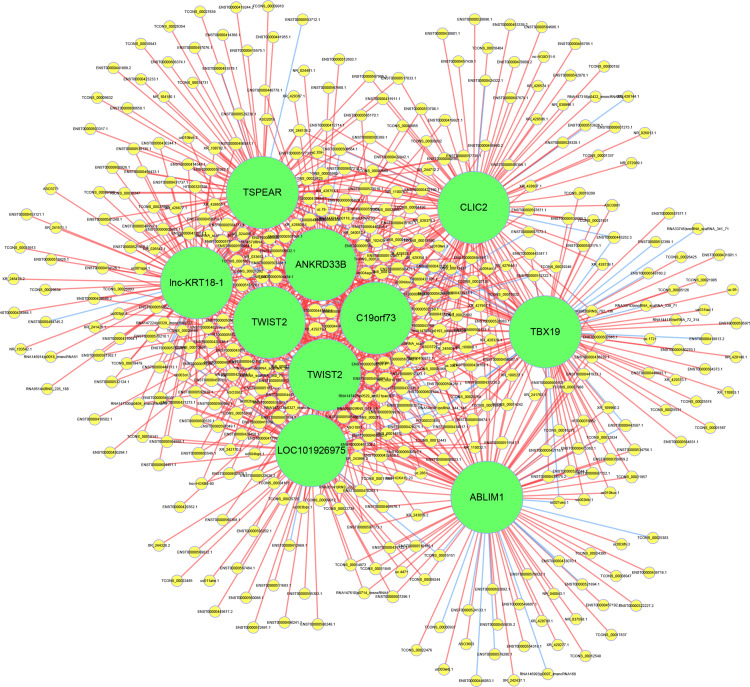
Construction of the lncRNA-mRNA coexpression network. The yellow circles represent lncRNAs, and the green circles represent mRNAs. The size of the circle is determined by the number of other genes that interact with this gene. The red line represents a positive correlation, and the blue line represents a negative correlation.

To evaluate the interactions among the key differentially expressed mRNAs and lncRNAs, a co-expression network was constructed. The results showed that lnc-KRT18-1, TSPEAR, CLIC2, ANKRD33B, TWIST2, C19orf73, TBX19, TWIST2, LOC101926975, and ABLIM1 had the highest number of interactions, which revealed the vital roles of these genes in the pathogenesis of OPLL (Figure 5).

### Target Prediction of lncRNAs

**FIGURE 6:**
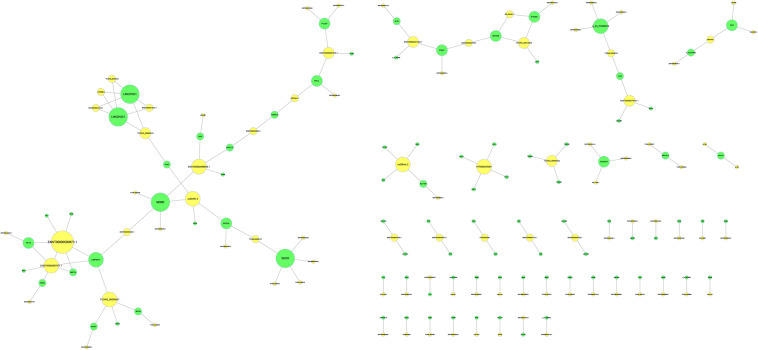
Target prediction of lncRNAs. The yellow circles represent lncRNAs, and the green circles represent the target genes. The size of the circle is determined by the number of other genes that interact with this gene.

To predict the potential target mRNAs of the differentially expressed lncRNAs, cis-acting lncRNA prediction and trans-acting lncRNA prediction were used, and gene target networks were created. In the potential target network, MDM2 was the most important target for the lncRNAs since MDM2 had the most interactions with lncRNAs. The gene target network is shown in Figure 6.

### Transcription Factor Prediction of lncRNAs

**FIGURE 7:**
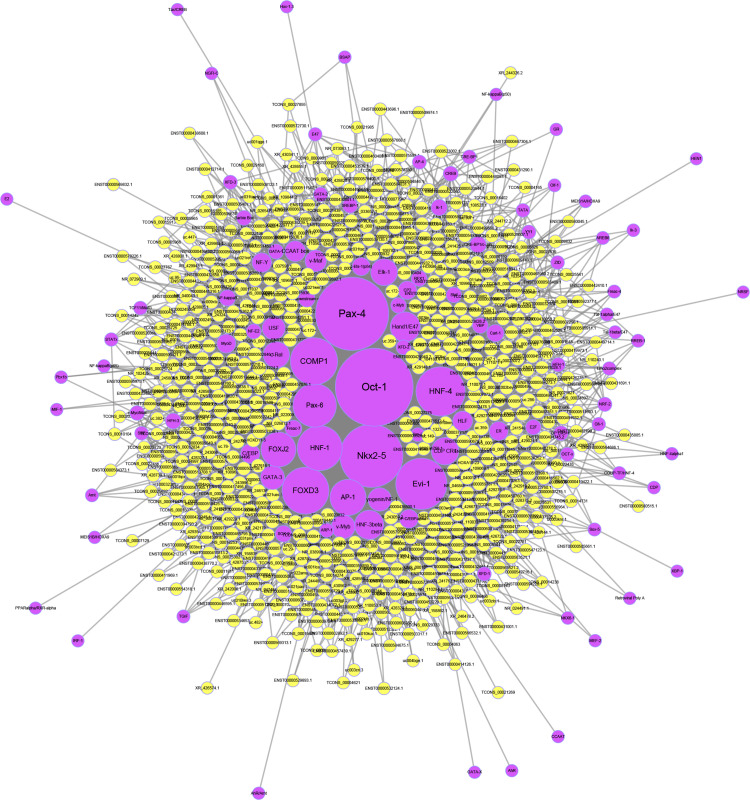
Transcription factor prediction of lncRNAs. The yellow circles represent lncRNAs, and the purple circles represent transcription factors. The size of each circle is determined by the number of other genes that interact with this gene.

To predict potential transcription factors of the differentially expressed lncRNAs, the Match-1.0 Public transcription factor prediction tool was used. As shown in Figure 7, Oct-1 was the most important transcription factor in the pathogenesis of OPLL since it had the highest number of interactions.

## Discussion

In this study, we performed microarrays to identify a number of lncRNAs and mRNAs aberrantly expressed in OPLL MSCs when they differentiated into osteoblasts. The cellular events and biological pathways involved in the abnormal osteogenic differentiation of OPLL MSCs were identified by GO and KEGG analyses. We further investigated the interactions between the aberrantly expressed lncRNAs and mRNAs in OPLL MSCs and identified core regulatory factors by constructing a co-expression network. Finally, we predicted the targets and transcription factors of the lncRNAs. Our results provide a basis for further understanding the role and mechanism of lncRNAs in the pathogenesis of OPLL.

Ectopic ossification in spinal ligament tissues is the chief characteristic of OPLL, and its progression leads to serious neurologic impairment (Xu et al., 2016). In recent years, the etiology of OPLL has been extensively investigated (Inamasu et al., 2006; Furukawa, 2008). Yoshifumi Harada suggested an increase in the osteogenic potential of MSCs from OPLL patients, which may be a pathogenic factor in OPLL (Harada et al., 2014; Imagama et al., 2017). Gentaro Kumagai showed that the MSCs from a spinal ossification mouse model had a higher osteogenic potential than those from normal mice (Liu X. et al., 2017). Our results confirmed that MSCs from OPLL patients had an increase in the osteogenic potential. However, the underlying mechanism of MSCs participating in the ectopic ossification of OPLL remains unclear.

Emerging evidence has revealed that lncRNAs can regulate osteogenic differentiation (He et al., 2018; Li J. et al., 2018; Tang Z et al., 2018). With the development of high-throughput technologies, an in-depth examination of the non-coding genome can be achieved (Kopp and Mendell, 2018). In our study, aberrantly expressed lncRNAs and mRNAs in the osteogenic differentiation of OPLL MSCs were identified by microarrays and confirmed by qRT-PCR assays. The results of the qRT-PCR assays were almost in complete agreement with the microarray data, further confirming the high credibility of the microarray data analysis. Among these aberrantly expressed lncRNAs and mRNAs, we found that ENPP1 was down-regulated in OPLL. A previous study has showed that Enpp1 knockout mouse is a model of OPLL (Saito et al., 2011), which supports our finding. Further studies are needed to explore the functions of other aberrantly expressed lncRNAs and mRNAs in OPLL. LncRNA expression profiles are important symbols for many diseases, including laryngeal cancer, systematic lupus erythematosus, rheumatoid arthritis, lung adenocarcinoma and intrahepatic cholangiocarcinoma (Zhang et al., 2016; Yang et al., 2017; Wang et al., 2018; Liu H. et al., 2019). Together with previous studies, our study highlights the importance of lncRNAs in the development of diseases.

To investigate the molecular function and pathways involved in the osteogenic differentiation of OPLL MSCs, GO and KEGG analyses were performed based on the differentially expressed mRNAs. KEGG analysis demonstrated that the p53, JAK-STAT and PI3K/Akt signaling pathways may be involved in the abnormal osteogenic differentiation of OPLL MSCs. A recent study showed that the activation of the JAK2-STAT3 pathway and the PI3K/Akt signaling pathway promoted osteogenic differentiation in OPLL (Chen et al., 2018). In addition, many researchers have found that the p53 signaling pathway mediates the osteogenic differentiation of MSCs (Yoon et al., 2016; Haffner-Luntzer et al., 2018). However, there are no studies on the role of the p53 signaling pathway in OPLL. Our results revealed the importance of the p53 signaling pathway in OPLL, and further studies need to explore its function in regulating the osteogenic differentiation of OPLL.

To identify the core regulatory factors involved in the osteogenic differentiation of OPLL, we constructed a lncRNA-mRNA co-expression network. Our results indicated that lnc-KRT18-1, TSPEAR, CLIC2, ANKRD33B, TWIST2, C19orf73, TBX19, TWIST2, LOC101926975, and ABLIM1 may play a central role in regulating the osteogenic differentiation of OPLL MSCs. There are no studies reporting their significant roles in the osteogenic differentiation of OPLL, and further experiments are needed.

To further investigate the regulatory mechanism of the aberrantly expressed lncRNAs, we predicted the potential targets and transcription factors of the lncRNAs. In the potential target network, MDM2 was the most important target for the lncRNAs since MDM2 had the most interactions with lncRNAs. Previous studies showed that osteoblast differentiation was regulated by the MDM2-p53 signaling pathway (Jin et al., 2017). Together with KEGG analysis, we proposed that the MDM2-p53 signaling pathway may play an important role in the osteogenic differentiation of OPLL. In addition to downstream target gene prediction, we also predicted the upstream transcription factors of the lncRNAs. We found that Oct-1 was the most important transcription factors since it had the highest number of interactions. Recently, Anirudha Karvande demonstrated that Oct-1 promoted osteoblast differentiation (Karvande et al., 2018). Therefore, we regard Oct-1 as an important transcription factor for lncRNAs in OPLL, and further studies are necessary to confirm its role in OPLL.

In conclusion, we detected the aberrantly expressed lncRNAs and mRNAs in the osteogenic differentiation of OPLL MSCs compared to HD MSCs and identified potential regulatory mechanisms with bioinformatic analyses. We aimed to reveal the role of lncRNAs in the abnormal osteogenic differentiation of OPLL. These results may help illuminate the pathogenesis of OPLL and provide new ideas for the diagnosis and treatment of OPLL. However, there are some limitations in our research. For example, we did not confirm the concrete functions of the aberrantly expressed lncRNAs in OPLL. Further studies are needed to explore the role of these aberrantly expressed lncRNAs in the osteogenic differentiation of OPLL MSCs.

## Data Availability Statement

Details of the microarray data can be found on the GEO website (https://www.ncbi.nlm.nih.gov/geo/query/acc.cgi?acc=GSE153829).

## Ethics Statement

This study was approved by the Ethics Committee of Sun Yat-sen Memorial Hospital, Sun Yat-sen University. The MSCs used in this study were obtained from the Center for Biotherapy, Sun Yat-sen Memorial Hospital, Sun Yat-sen University.

## Author Contributions

ZC, WL, KC, PW, ZX, JL, ML, SC, GY, ZL, ZS, and MM performed the trials. ZC and WL analyzed the data. YW and HS contributed the reagents. ZC wrote the manuscript. YW and HS revised the manuscript. All authors contributed to the article and approved the submitted version.

## Conflict of Interest

The authors declare that the research was conducted in the absence of any commercial or financial relationships that could be construed as a potential conflict of interest.
