# Supplementary material for: Aberrantly Expressed lncRNAs and mRNAs of Osteogenically Differentiated Mesenchymal Stem Cells in Ossification of the Posterior Longitudinal Ligament
Source: Front Genet. 2020 Aug 7;11:896. doi: 10.3389/fgene.2020.00896 (PMC7426401; doi:10.3389/fgene.2020.00896)
Supplement: Supplementary file 1 [file Table_1.DOCX]

**Supplementary Table S1:** **Details of the study subjects**

|  | Healthy donors | OPLL patients |
| --- | --- | --- |
| Number | 30 | 30 |
| Age, years | 55.7±7.7 | 55.1±7.9 ^a^ |
| Male, n (%) | 18 (60.0%) | 19 (63.73%) ^a^ |
| BMI | 22.9±3.2 | 23.2±2.9^a^ |

OPLL, ossification of the posterior longitudinal ligament; BMI, body mass index. ^a^, indicates *P*<0.05 compared to the healthy donor group. Data are presented as the mean ± SD.

**Supplementary Table S2: primer sequences used for qRT-PCR**

| Gene Name | Forward Primer | Reverse Primer |
| --- | --- | --- |
| GAPDH | AAGGTGAAGGTCGGAGTCAA | AATGAAGGGGTCATTGATGG |
| p11878 | CACAGCGTGTGGTACTCTCTT | TTCTGTTCTGTTCCCACTGGC |
| p23569 | ACAGACCATGTTCCGTGCTC | GCTCTGTCTCTCGTTGCTGT |
| p22941 | CCGGCTATTTTGGAGATTGAAA | GCGGTAGAATAGAAATGACAGATG |
| p20197 | CTTCACCGGAAGACTCGGAA | ATCCTGCACAGGGTGGCATA |
| p13631 | AGAGGGTAGGTATCCTGTAGCC | CCTTAGCTTATGCTCCCCCT |
| p13632 | ATCCGGCTATTTTGGAGATTGA | TGCGGTAGAATAGAAATGACAGATG |
| p26842 | AGCGCTAGCTTCACTTGATCT | AAAGAGGGGGAAACAATTTGC |
| p1957 | AGCATGTGGCAGAGAACACA | TGGGTTTGATGTGACTTGGC |
| p8129 | CTGCCTCAAAACGACGTCAC | AAAGAGACCCCCAAGGAGGA |
| p4982 | GGACTTCCCTGAGATTTGGCT | ATGAGACGTCAGCTTTCTCCC |
| CST2 | AGGAGGACAGGATAATCGAGGG | CGCTGTACCCGCTCATCATT |
| HAPLN1 | TCTGGTGCTGATTTCAATCTGC | TGCTTGGATGTGAATAGCTCTG |
| CLSTN2 | GTGCTCAACAAGACATCAGGA | CCTTCACCTGTATATGGACCACG |
| NDUFA4L2 | ATGATCGGCTTAATCTGCCTG | TCCGGGTTGTTCTTTCTGTCC |
| CST1 | ATCCCGGGTGGCATCTATAAC | AGCGGACGTCTGTAGTAGTCATC |
| OLFM2 | TGGGTGCCTACGGGTATGAG | GGCCATCGTGTCAGTCATCC |
| CYP26B1 | GGCAACGTGTTCAAGACGC | TGCTCGCCCATGAGGATCT |
| ADH1C | GGTTTTGAGTGCTGGTTCGG | TTTTAAGCCACACCCGTCGT |
| ADH1B | AAGACTCACAGTCTGCTGGTG | GCATTTGATTACTTTTCCTGCTGTG |
| MMP13 | CCAGACTTCACGATGGCATTG | GGCATCTCCTCCATAATTTGGC |

**Supplementary Table S3:** **The validation result about the microarray and qRT-PCR**

| Gene | Microarray | qRT-PCR |
| --- | --- | --- |
| p11878 | up | up |
| p23569 | up | up |
| p22941 | up | up |
| p20197 | up | up |
| p13631 | up | up |
| p13632 | up | up |
| p26842 | down | down |
| p1957 | up | up |
| p8129 | up | up |
| p4982 | up | up |
| CST2 | up | up |
| HAPLN1 | down | down |
| CLSTN2 | up | up |
| NDUFA4L2 | up | up |
| CST1 | up | up |
| OLFM2 | up | up |
| CYP26B1 | up | up |
| ADH1C | up | up |
| ADH1B | up | up |
| MMP13 | down | down |
